# Carnrick’s Food

**Published:** 1888-09

**Authors:** 


THE conditions formulated by the Committee on Infants' Foods at the American Medical Association are approximated more nearly by Carnrick's Food than by any other with which we are familiar.—*Editorial note in Philadelphia Medical Times, June 1, 1888.*

